# Predicting In-Hospital Acute Heart Failure Worsening in the Oldest Old: Insights from Point-of-Care Ultrasound

**DOI:** 10.3390/jcm12237423

**Published:** 2023-11-30

**Authors:** Tessa Mazzarone, Virginia Morelli, Andrea Giusti, Maria Giovanna Bianco, Lorenzo Maccioni, Cristina Cargiolli, Daniela Guarino, Agostino Virdis, Chukwuma Okoye

**Affiliations:** 1 Geriatrics Unit, Department of Clinical and Experimental Medicine, University of Pisa, 56126 Pisa, Italy; 2 Department of Neurobiology, Care Sciences and Society, Aging Research Center, Karolinska Institutet and Stockholm University, 11419 Stockholm, Sweden; 3 Department of Medicine and Surgery, University of Milano-Bicocca, 20126 Milano, Italy

**Keywords:** heart failure, older adults, ultrasound, outcomes, pleural effusion

## Abstract

The decompensation trajectory check is a basic step to assess the clinical course and to plan future therapy in hospitalized patients with acute decompensated heart failure (ADHF). Due to the atypical presentation and clinical complexity, trajectory checks can be challenging in older patients with acute HF. Point-of-care ultrasound (POCUS) has proved to be helpful in the clinical decision-making of patients with dyspnea; however, to date, no study has attempted to verify its role in predicting determinants of ADHF in-hospital worsening. In this single-center, cross-sectional study, we consecutively enrolled patients aged 75 or older hospitalized with ADHF in a tertiary care hospital. All of the patients underwent a complete clinical examination, blood tests, and POCUS, including Lung Ultrasound and Focused Cardiac Ultrasound. Out of 184 patients hospitalized with ADHF, 60 experienced ADHF in-hospital worsening. By multivariable logistic analysis, total Pleural Effusion Score (PEFs) [aO.R.: 1.15 (CI95% 1.02–1.33), *p* = 0.043] and IVC collapsibility [aO.R.: 0.90 (CI95% 0.83–0.95), *p* = 0.039] emerged as independent predictors of acute HF worsening after extensive adjustment for potential confounders. In conclusion, POCUS holds promise for enhancing risk assessment, tailoring diuretic treatment, and optimizing discharge timing for older patients with ADHF.

## 1. Introduction

Heart failure (HF) stands as a significant contributor to hospital admissions in individuals aged 65 and above. Its prevalence in developed countries is approximately 1–2%, a figure that escalates to 10% when focusing on individuals over 70 years of age [1,2].

Patients admitted with heart failure (HF) face a substantial 20% to 30% risk of mortality within the first year of admission [3]. Although a swift recovery leading to discharge within 4 to 5 days is a frequent occurrence among HF patients, any observed deterioration during their hospital stay may signal a noteworthy alteration in the overall trajectory of their condition. The American College of Cardiology advocates for a timely recognition of clinical trajectories in acute heart failure. It is possible to define three main in-hospital trajectories: improving towards target, stalled after initial improvement, or not improved/worsening [3].

These trajectories translate into different management strategies throughout the hospitalization and post-discharge, by optimizing guideline-based treatment, proceeding with further diagnostic investigation, or increasing diuretic therapy. Ensuring the precision with which the correct trajectory is intercepted is of pivotal importance, especially in the case of elderly patients who carry an elevated risk of experiencing disease recurrence or short-term hospitalization attributed to HF [4]. However, due to their atypical clinical presentations and their complexity caused by coexisting comorbidities and frailty, trajectory checks can be challenging in the oldest old affected by acute decompensated heart failure (ADHF).

Point-of-Care Ultrasound (POCUS) involves non-radiologist healthcare providers performing targeted ultrasound at the patient’s bedside to facilitate a rapid diagnosis [5].

POCUS has proven to be a helpful non-invasive tool used for the detection and quantification of pulmonary congestion in both ambulatory and hospitalized patients [6,7,8]. Moreover, several studies suggest that Lung Ultrasound (LUS) outperforms chest radiography in detecting pulmonary edema and it is comparable to chest Computed Tomography in identifying lung pathology in patients with acute respiratory failure [9,10,11]. Nevertheless, only a limited number of studies have endeavored to assess the effectiveness of POCUS in identifying early determinants of in-hospital worsening of ADHF [12]. Based on these foundations, the primary objective of this study was to examine the potential of POCUS as a predictive tool for the trajectory of ADHF. Specifically, the study aimed to identify the ultrasound early determinants of in-hospital clinical deterioration.

## 2. Materials and Methods

In this single center, cross-sectional study, we consecutively enrolled patients aged 75 years or older hospitalized with ADHF in the Geriatrics Unit of a tertiary care hospital. The exclusion criteria were: (a) patients unable to provide their written or oral consent; (b) patients with invasive ventilation at hospital admission. A panel of clinicians adjudicated the diagnosis of congestive heart failure based on clinical symptoms, signs, chest X-ray film results, echocardiographic findings, and therapy at admission in line with recent international guidelines [1]. All the patients underwent physical examination, complete blood tests, and a comprehensive geriatric assessment (CGA) [13], including a cognitive evaluation using the Short Portable Mental Status Questionnaire (SPMSQ) [14], level of autonomy in terms of independence in the performance of basic (ADL) [15], and instrumental (IADL) [16], activities of daily living. Frailty assessment was evaluated through the Clinical Frailty Scale (CFS) [17]. The risk of malnutrition was assessed through the Mini Nutritional Assessment-Short Form (MNA-SF) [18] and Body Mass Index (BMI).

Furthermore, patients underwent a diagnostic examination early during hospitalization (within the first 48 h) with bedside Point-of-Care Ultrasound (POCUS) including a Lung Ultrasound, a Focused Cardiac Ultrasound, pleural effusion score (PEFs) [19], and Inferior Vena Cava (IVC) assessment. A convex and a linear covered probe, (3.5 to 7.5 MHz), were used for chest ultrasound examination, a phased array probe with bandwidth of 1.7–3.8 MHz was utilized for focused ultrasound (Esaote Medical System, Florence, Italy). For patients with severe mobility limitations, two operators were concomitantly involved, according to current guidelines [20]. 8-zone LUS was performed (anterosuperior, anteroinferior, lateral superior, lateral inferior) at scanning depths of 10, 12, and 16 cm; the evaluation involved assessing the presence of B-lines, and a score ranging from 0 to 5 was assigned per field based on the quantity observed (max 20 per hemithorax). PEFs was assessed by positioning the probe longitudinally (perpendicular to the ribs) in an intercostal space in the posterior axillary line with the patient in a semi-recumbent position so that the liver or spleen, diaphragm, and lung as well as the spine were visualized in one image. PEFs value ranging from 0 to 4 points for each hemithorax and to estimate the cumulative fluid burden the score of both hemi-thoraces were summed for an overall range of 0–8.

Apical four-chamber view, apical five-chamber view, parasternal long-axis view, parasternal short-axis view, and subcostal four-chamber view were obtained. The final FOCUS report contained information on Left Ventricle: systolic function (assessed with the biplane method of disks (modified Simpson’s rule), myocardial dyskinesia, size, myocardial thickness. Right ventricle: size, signs of acute chronic pressure overload, valves aortic and mitral valve pathology, volume status: pericardial effusion, inferior vena cava (IVC) overload. The IVC diameter was measured during inspiration and expiration to assess collapsibility. Left ventricular function was also estimated via Mitral Valve E-Point Septal Separation (EPSS). The EPSS is an easy-to-perform metric employed in focused echocardiography to evaluate left ventricular function. This measurement is derived from the parasternal long-axis view (PLAX) of the heart, specifically utilizing M-mode echocardiography focused on the distal tip of the anterior leaflet of the mitral valve. The M-mode trace of the mitral valve waveform reveals two distinct peaks: the first, larger peak, known as the “E-point,” signifies the maximal opening of the mitral valve during early left ventricular diastole. The second, smaller peak, termed the “A-point,” corresponds to atrial contraction later in left ventricular diastole. In a normally functioning heart, the mitral valve should open with the leaflet making contact or closely approaching the interventricular septum at the E-point. The distance between the E-point and the interventricular septum represents EPSS. A diminished EPSS indicates a healthy heart, characterized by minimal separation between the mitral valve and the septum during early diastole. An EPSS measurement exceeding 7 mm is indicative of LVEF. Notably, an EPSS equal to or greater than 13 mm correlates with severely impaired cardiac function, suggesting an estimated LVEF of 35% or less [21].

Clinical aggravation was defined as a composite endpoint of:

- at least a 10% reduction in P/F ratio
- an increase on dosage of diuretic therapy,
- onset of pulmonary edema during hospitalization, and
- in-hospital death.

The 30 post-discharge mortality rate was assessed by phone interview.

### Statistical Analysis

A statistical analysis was performed with IBM SPSS Statistic (IBM SPSS Statistic version 27.0 lnk IBM Corporation, Armonk, NY, USA, and its licensor 1989–2020) and RStudio (RStudio Team: Integrated Development for R. RStudio, PBC, Boston, MA, USA). Continuous variables are presented as median (interquartile range, IQR) or mean (standard deviation, SD), and categorical variables as counts and percentages. Mann–Whitney and chi-square tests were used for multiple comparisons. A multivariable logistic regression was performed to evaluate the association between statistically significant variables of the univariable model and in-hospital worsening using a priori selected model covariates on the basis of clinical considerations. Covariates included age, sex, main comorbidities, BMI, and LVEF. Estimated odds ratios (O.R.s) with 95% confidence intervals (CIs) were obtained. As a secondary analysis, we divided patients into three groups on the basis of the sum of baseline B-lines in tertiles in 8 zones: Tertile 1: <7 B-lines; Tertile 2: 7–15 B-lines; Tertile 3: ≥15 B-lines. In a subgroup of 30 patients in whom LUS was performed blindly by the two Italian Society for Ultrasound in Medicine and Biology-certified operators (C.O., T.M), the interobserver agreement was calculated.

## 3. Results

Out of 184 patients hospitalized with ADHF enrolled in the study (mean [SD], 86.8 [5.9] years), 60 (32.6%) experienced HF in-hospital worsening. No differences were found between patients with HF worsening and controls in terms of sex, mean age, body weight, left ventricular ejection fraction, and the number of comorbidities. Regarding comorbidities, patients with in-hospital worsening had a higher prevalence of atrial fibrillation (AF) [78.6% vs. 56.9%, *p* = 0.006] and coronary artery disease (CAD) [48.2% vs. 29.3%, *p* = 0.001], compared to their counterparts.

Patients with HF worsening had no differences in terms of frailty detected by CFS [median CFS: 5.5 (IQR = 3) vs. 6 (IQR = 3), respectively, *p* = 0.55]. Moreover, patients with clinical worsening showed a higher degree of congestion in terms of pleural effusion estimated by PEFs [median cumulative PEFs: 4 (IQR = 5) vs. 2 (IQR = 4), respectively, *p* = 0.005] and reduction of Inferior Vena Cava collapsibility [delta IVC mean: 6 (SD = 4.9) vs. 8.9 (IQR = 5.2), respectively, *p* = 0.002] than their counterparts. No differences were detected in terms of B-line numbers. Moreover, no significant differences in terms of outcomes were found across B-line tertiles (*p* = 0.59). Interobserver agreement for POCUS, calculated in a subsample of patients, was high (k = 0.86) (Table 1).

**Figure 1:**
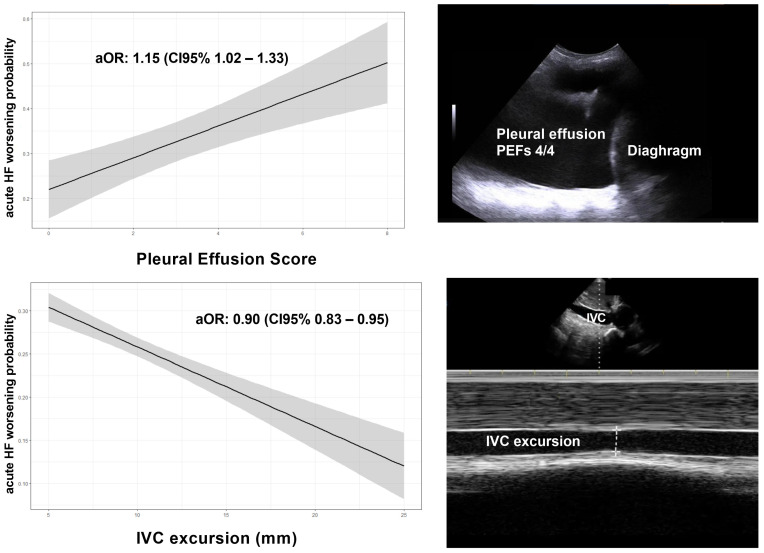
Relationship between Pleural Effusion Score, IVC and acute HF worsening probability.

As shown in Table 2, by multivariate logistic analysis, total PEFs [adjusted O.R.: 1.15 (CI95% 1.02–1.33), *p* = 0.043], and IVC collapsibility [adjusted O.R.: 0.90 (CI95% 0.83–0.95), *p* = 0.039] resulted independently associated with HF in-hospital worsening after extensive adjustment for age, sex, main comorbidities, BMI and left ventricular ejection fraction (Figure 1).

## 4. Discussion

In the present study, a higher degree of pleural effusion and a reduced IVC collapsibility evaluated by POCUS proved to be strong independent predictors of HF in-hospital worsening in older patients with acutely decompensated heart failure. Moreover, the results suggest that POCUS may provide clinicians with a non-invasive tool for accurately predicting ADHF and deterioration in older patients.

Indeed, previous studies showed how the integration of POCUS with clinical assessment for the diagnosis of ADHF in the emergency department appears to be more accurate than standard diagnostic approaches based on CXR and NT-proBNP [8,22]. Furthermore, in patients admitted for ADHF, the ongoing presence of congestion, as identified by LUS before discharge, has been proven to serve as a crucial predictor for both HF-related rehospitalizations and mortality [12,23,24]. In the present investigation, we further demonstrated the utility of POCUS in the trajectory check of acute HF. This study emphasizes the significance of routinely monitoring and evaluating the pleural effusion and the IVC collapsibility in older patients with acute heart failure.

Furthermore, POCUS could aid clinicians in tailoring HF diuretic therapy during hospitalization, improving discharge timing. The concept of a trajectory check stresses the importance of stepping back to gain perspective on where the patient stands and in which direction the patient is headed. In our study, the odds of adverse outcomes increased by 15% for every 1-unit increase in the total PEFs and reduced by 10% for every 1-unit increase in IVC collapsibility. Even if its role in the diagnosis of acute heart failure is well recognized, the prognostic relevance of pleural effusion in older patients has not been investigated systematically [25]. Nonetheless, several studies have successfully linked its presence to an increased risk of mortality [26,27,28]. Moreover, patients with bilateral pleural effusions demonstrated a notably higher risk of mortality when compared to those with unilateral effusions. Numerous studies have demonstrated that thoracic ultrasound outperforms chest radiography in terms of sensitivity and specificity when detecting pleural effusions [29,30,31], and our findings strongly support its potential utility as an indicator of in-hospital deterioration. On the other hand, as it is well established, the IVC dilates with an increase in right atrial pressure, making it a potential indicator of HF severity that is not dependent on left ventricular ejection fraction (LVEF) [24]. Previous research has demonstrated that POCUS assessment can identify individuals with chronic heart failure at risk of unfavorable outcomes by detecting IVC diameter enlargement, irrespective of their LVEF status [23].

The effectiveness of POCUS in addressing acute dyspnea and HF management is widely recognized [6], yet the predictive significance of early POCUS indicators for in-hospital deterioration in patients with ADHF has not been systematically explored. Previous studies have primarily employed time-consuming 28-zone imaging protocols [32,33]. However, Platz et al. recently demonstrated that a four-zone LUS evaluation can effectively detect pulmonary congestion and that a higher count of B-lines during early admission can identify patients at an elevated risk of in-hospital events [12], encouraging a simplified approach.

Surprisingly, B-lines did not prove to be significant predictors of in-hospital deterioration among the geriatric population with acute decompensated heart failure (ADHF). This finding runs counter to various studies that have consistently highlighted the quantification of B-lines as a valuable predictor of heart failure recurrence and mortality in hospitalized patients with heart failure [12,33,34]. This discrepancy may be attributed to the limited specificity of B-lines themselves. While the detection of a B-pattern on LUS exhibits high sensitivity for diagnosing pulmonary edema, its specificity is notably low. This lack of specificity is particularly evident in the oldest old, where underlying lung diseases could potentially influence LUS findings. Multiple B-lines typically indicate an interstitial syndrome, encompassing conditions such as interstitial lung diseases (e.g., fibrosis), pulmonary manifestations of connective tissue diseases, and pneumonia [35,36,37]. Pneumonia, for instance, is frequently observed in elderly patients admitted for acute HF [38,39] and can confound the interpretation of B-lines on LUS due to the combination of cardiogenic and inflammatory edema. Furthermore, occasional B-lines (up to two) can be present in normal lungs, typically at the lung bases. Additionally, patients may exhibit clinical improvement even if they continue to display B-lines during LUS evaluations, irrespective of their ADHF trajectory. Concerning comorbidities, we observed that patients affected by atrial fibrillation (AF) or coronary artery disease (CAD) presented an increased risk of in-hospital worsening.

However, at least two limitations of the study need to be acknowledged. Firstly, as a single-center investigation, further multicenter studies are warranted to validate the prognostic significance of PEFs and reduced IVC collapsibility evaluated by POCUS as strong independent predictors of acute HF in-hospital worsening; however, the clinical uniformity of the cohort and the clear protocol represent strengths of our investigation. Secondly, POCUS is a highly operator-dependent imaging modality with several knobology variables that are under the control of the operator. Knobology is a terminology that describes the manipulation of ultrasound knobs and system controls to obtain the best image possible from diagnostic ultrasound. The inadequate use of knobology variables may impair image quality and can result in misdiagnosis. The simple change of positioning of the probe with respect to the curvature of the patient’s chest can modify the perceptual semi-quantitative evaluation of B-lines, that may, therefore, be very variable in different intra- and inter-operator examination. Additionally, the increase in the pleural line movement rate can modify the perception of B lines. However, the current study POCUS was performed by expert physicians yielding a high interobserver agreement.

In light of the current findings, a promising avenue for future research involves exploring the practical implications of a POCUS-directed therapy in optimizing outcomes for older patients with ADHF. Investigating whether tailored interventions guided by real-time POCUS assessments can lead to superior patient outcomes is a compelling area for further exploration. By evaluating the impact of POCUS-directed therapeutic strategies, researchers could assess the feasibility and efficacy of adjusting treatment plans based on immediate ultrasound findings. This approach may involve dynamic adjustments to diuretic therapy, discharge planning, and overall HF management. Investigating the implementation of POCUS as a guiding tool for therapeutic decision-making could not only enhance the precision of interventions but also potentially contribute to shorter hospital stays, reduced rates of readmission, and improved overall patient well-being. Additionally, such research endeavors could delve into establishing standardized protocols for POCUS-directed therapy, providing a framework for clinicians to integrate ultrasound findings seamlessly into their treatment algorithms. This approach may foster a more patient-centered, individualized care model, aligning with the evolving landscape of precision medicine in cardiovascular care. In conclusion, this study represents the initial documentation of both total pleural effusions and IVC collapsibility as significant indicators for predicting in-hospital deterioration among older patients admitted for ADHF. Recognizing predictors of in-hospital deterioration in geriatric patients with ADHF is crucial for clinicians, as it can lead to reduced hospitalization durations and better outcomes. Recent research has shown that extended hospital stays following HF hospitalization are linked to higher rates of various types of readmissions [4]. POCUS can aid medical professionals in enhancing risk assessment and personalizing diuretic treatment for hospitalized heart failure patients, ultimately optimizing the timing of patient discharge.

## 5. Conclusions

POCUS has the potential to serve a significant role in monitoring pulmonary congestion throughout hospitalizations for ADHF and enhancing risk assessment by identifying early indicators of in-hospital deterioration. Poor IVC collapsibility and a higher amount of pleural effusion are markers of worsening acute heart failure during the first phase of hospitalization for ADHF.

## Figures and Tables

**Table 1:** Characteristics of study population.

	All Patients *n* = 184	Adverse Event *n* = 60	Controls *n* = 124	*p*-Value
Gender F (%)	67 (58)	25 (62.5)	49 (56.3)	0.95
Mean Age (years)	86.8 (5.9)	87.2 (5.9)	86.6 (5.8)	0.51
Mean BMI	24.6 (4.5)	24.3 (4.3)	24.4 (4.5)	0.85
Median CFS	6 (3)	5.5 (3)	6 (3)	0.55
Arterial Hypertension (%)	121 (66.2)	41 (69.6)	80 (64.6)	0.517
Atrial Fibrillation (%)	118 (63.9)	47 (78.5)	70 (56.8)	0.006
Coronary Artery Disease (%)	65 (35.4)	29 (48.2)	36 (29.3)	0.001
Diabetes Mellitus (%)	53 (29.0)	15 (25.0)	38 (31.0)	0.414
Peripheral Artery Disease (%)	49 (26.7)	15 (25.0)	34 (27.5)	0.720
Cancer (%)	37 (20.4)	7 (12.4)	30 (24.1)	0.076
Chronic Kidney Disease (%)	58 (31.9)	21 (35.7)	37 (30.1)	0.46
Chronic Obstructive Pulmonary Disease (%)	48 (26.1)	19 (32.1)	28 (23.2)	0.21
History of Stroke (%)	33 (18.0)	11 (17.8)	22 (18.1)	0.96
Median B-lines	12 (12)	13 (15.2)	11 (13)	0.18
PEFs cumulative	2 (5)	4 (5)	2 (4)	0.005
IVC max (mm)	17.7 (5.2)	18.2 (5.1)	17.2 (5.2)	0.27
IVC min (mm)	9.8 (7.2)	12.2 (7.5)	8.2 (6.9)	0.002
Delta IVC (mm)	7.8 (5.2)	6 (4.9)	8.9 (5.2)	0.002
EPSS (mm)	6.1 (3.2)	4.9 (2.4)	6.8 (3.5)	0.024
NT-proBNP max (ng/L)	14,430.7 (28,237.2)	15,504 (13,858.5)	14,525.2 (33,945.3)	0.84
NT-proBNP min (ng/L)	8616.3 (28,885.9)	7050.4 (7710.7)	9536.8 (35,647.2)	0.63
Serum Creatinine (mg/dL)	1.39 (0.71)	1.45 (0.75)	1.26 (0.59)	0.21
Hospital stays (days)	7 (3)	8 (3)	6.5 (3)	0.004
P/F at admission	311.3 (93.7)	296 (99.2)	318.5 (91.4)	0.15
HCO_3_^−^ admission	25.4 (4.4)	26.2 (4.5)	25 (4.2)	0.09
HCO_3_^−^ at discharge	29.3 (5.7)	29.7 (5.9)	29.2 (5.7)	0.58

Continuous variables are expressed as mean SD or median with IQR properly. BMI, Body Mass Index; CFS, Clinical Frailty Scale; PEFs, Pleural Effusion score; IVC, Inferior Vena Cava; EPSS, E-Point Septal Separation; NT, N-terminal; BNP, B-type Natriuretic Peptide; P/F, PaO_2_/FiO_2_.

**Table 2:** Predictors of acute HF worsening. Univariable and multivariable logistic analysis.

	Univariable		Multivariable	
	O.R. (95%CI)	*p*-Value	O.R. (95%CI)	*p*-Value
PEFs	1.18 (1.05–1.41)	0.027	1.15 (1.02–1.33)	0.043
IVC collapsibility	0.84 (0.78–0.90)	0.021	0.90 (0.83–0.95)	0.039

O.R.: odds ratio, PEFs: Pleural Effusion score, IVC, Inferior Vena Cava; Multivariable regression: age, sex, main comorbidities, BMI and left ventricular ejection fraction as covariates.

## Data Availability

The datasets used and/or analysed during the current study are available from the corresponding author on reasonable request.
